# Diacetato[*N*,*N*′-bis­(2-pyridylmethyl­idene)cyclo­hexane-1,2-diamine]manganese(II) hexa­hydrate

**DOI:** 10.1107/S160053680800353X

**Published:** 2008-02-06

**Authors:** In-Chul Hwang, Kwang Ha

**Affiliations:** a Department of Chemistry, Pohang University of Science and Technology, Pohang 790-784, Republic of Korea; b School of Applied Chemical Engineering, Research Institute of Catalysis, Chonnam National University, Gwangju 500-757, Republic of Korea

## Abstract

The asymmetric unit of the title compound, [Mn(C_2_H_3_O_2_)_2_(C_18_H_20_N_4_)]·6H_2_O, consists of a neutral Mn^II^ complex with six solvent water mol­ecules. In the complex, the Mn^2+^ ion is eight-coordinated in a distorted square-anti­prismatic environment by four N atoms from the tetra­dentate ligand *N*,*N*′-bis­(2-pyridylmethyl­idene)cyclo­hexane-1,2-diamine (bpic) and four O atoms from two acetate ligands. The compound displays inter­molecular O—H⋯O hydrogen-bond inter­actions to form various kinds of ring structures and cyclic water clusters.

## Related literature

For details of some other Mn(bpic) complexes, see: Hwang & Ha (2007 ▶); Lu *et al.* (2006 ▶); Schoumacker *et al.* (2003 ▶). For related literature, see: Bernstein *et al.* (1995 ▶).
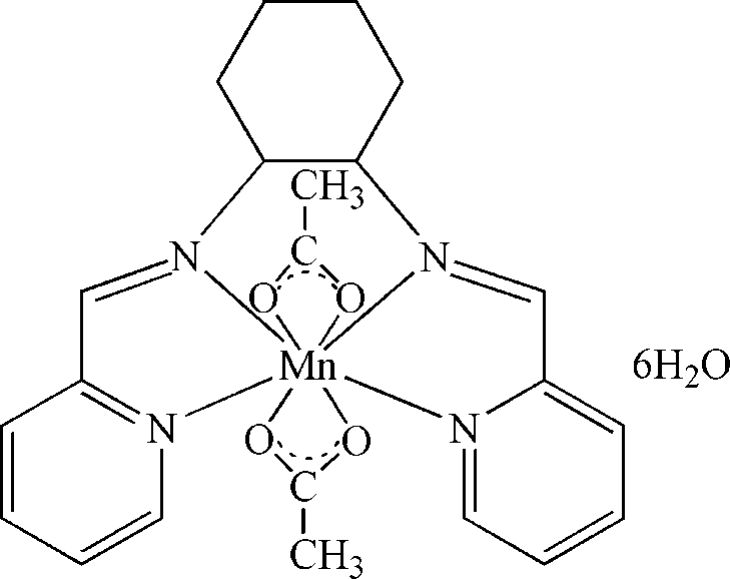


## Experimental

### 

#### Crystal data

[Mn(C_2_H_3_O_2_)_2_(C_18_H_20_N_4_)]·6H_2_O
*M* *_r_* = 573.50
Triclinic, 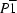

*a* = 8.5124 (5) Å
*b* = 11.6768 (7) Å
*c* = 15.1971 (10) Å
α = 79.049 (1)°
β = 85.195 (1)°
γ = 71.484 (1)°
*V* = 1405.83 (15) Å^3^
*Z* = 2
Mo *K*α radiation
μ = 0.53 mm^−1^
*T* = 243 (2) K
0.25 × 0.25 × 0.20 mm

#### Data collection

Bruker SMART 1000 CCD diffractometer
Absorption correction: multi-scan (*SADABS*; Bruker, 2000 ▶) *T*_min_ = 0.824, *T*_max_ = 0.900
11880 measured reflections
5710 independent reflections
4641 reflections with *I* > 2σ(*I*)
*R*_int_ = 0.015

#### Refinement

*R*[*F* ^2^ > 2σ(*F* ^2^)] = 0.040
*wR*(*F* ^2^) = 0.111
*S* = 1.02
5710 reflections
348 parameters
H-atom parameters constrained
Δρ_max_ = 0.44 e Å^−3^
Δρ_min_ = −0.20 e Å^−3^

### 

Data collection: *SMART* (Bruker, 2000 ▶); cell refinement: *SAINT* (Bruker, 2000 ▶); data reduction: *SAINT*; program(s) used to solve structure: *SHELXS97* (Sheldrick, 2008 ▶); program(s) used to refine structure: *SHELXL97* (Sheldrick, 2008 ▶); molecular graphics: *ORTEP-3* (Farrugia, 1997 ▶) and *PLATON* (Spek, 2003 ▶); software used to prepare material for publication: *SHELXL97*.

## Supplementary Material

Crystal structure: contains datablocks glonal, I. DOI: 10.1107/S160053680800353X/dn2316sup1.cif

Structure factors: contains datablocks I. DOI: 10.1107/S160053680800353X/dn2316Isup2.hkl

Additional supplementary materials:  crystallographic information; 3D view; checkCIF report

## Figures and Tables

**Table 1:** Hydrogen-bond geometry (Å, °)

*D*—H⋯*A*	*D*—H	H⋯*A*	*D*⋯*A*	*D*—H⋯*A*
O1*W*—H1*WA*⋯O3	0.922	1.793	2.705 (2)	169
O1*W*—H1*WB*⋯O2*W*	0.728	2.054	2.770 (3)	168
O2*W*—H2*WA*⋯O3*W*	0.724	2.523	2.738 (3)	100
O2*W*—H2*WB*⋯O6*W*^i^	0.862	1.975	2.835 (3)	176
O3*W*—H3*WA*⋯O1*W*^ii^	0.846	1.972	2.807 (3)	169
O3*W*—H3*WB*⋯O5*W*^iii^	0.929	1.915	2.821 (3)	165
O4*W*—H4*WA*⋯O2^iv^	0.868	1.986	2.845 (2)	170
O4*W*—H4*WB*⋯O4	0.875	1.892	2.759 (2)	171
O5*W*—H5*WA*⋯O4*W*	0.839	1.993	2.821 (3)	169
O5*W*—H5*WB*⋯O6*W*^v^	0.818	2.281	2.937 (3)	138
O6*W*—H6*WA*⋯O1	0.933	1.820	2.741 (2)	169
O6*W*—H6*WB*⋯O1*W*	0.916	1.915	2.803 (3)	163

Symmetry codes: (i) 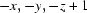; (ii) 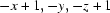; (iii) 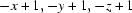; (iv) 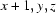; (v) 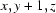.

**Fig. 1.**
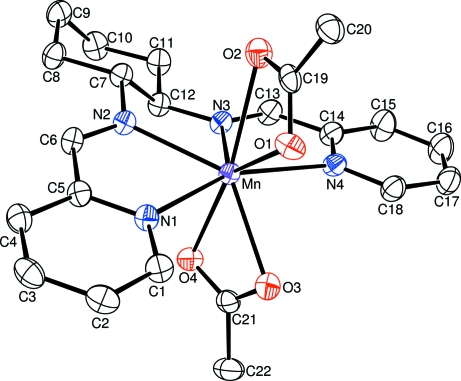
The structure of the title compound with the numbering scheme. Displacement ellipsoids are drawn at the 30% probability level. H atoms and the solvent H2O molecules have been omitted for clarity.

**Fig. 2.**
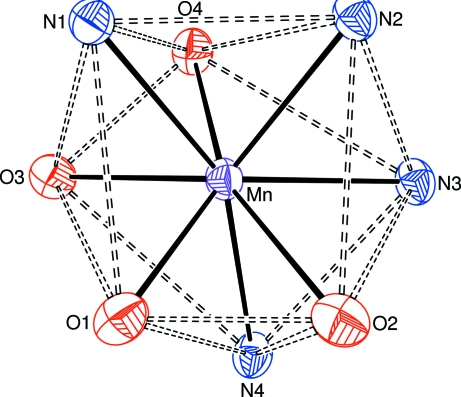
View of the distorted square antiprismatic geometry around the eight-coordinated Mn atom. Dashed lines (no bonds) are used for the clear representation of the structure.

**Fig. 3.**
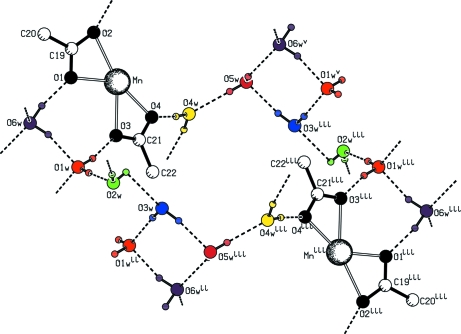
The structure and hydrogen-bond connectivity in the title compound [Symmetry codes: (ii) -x + 1, -y, -z + 1, (iii) -x + 1, -y + 1, -z + 1, (v) x, y + 1, z]. Hydrogen-bond interactions are drawn with dashed lines.

## supplementary crystallographic information

### Comment

The crystal structure of the title compound, [Mn(CH_3_CO_2_)_2_(C_18_H_20_N_4_)].6(H_2_O), consists of a neutral Mn^II^ complex with six solvent water molecules (Fig. 1). In the complex, the Mn^2+^ ion is eight-coordinated in a distorted square antiprismatic environment by four N atoms from the tetradentate ligand *N*,*N*'-bis(2-pyridylmethylidene)cyclohexane-1,2-diamine (bpic) and four O atoms from two acetate anion ligands (Fig. 2). The four N atoms and the four O atoms lie approximately on respective coordination planes with the largest deviations 0.223 Å (N3) and 0.219 Å (O1) from the respective least-squares planes, and the dihedral angles between these planes is 87.67 (3)°. The Mn—N(pyridyl) bonds (2.4166 (16) and 2.3899 (17) Å) are slightly longer than the Mn—*N*(imine) bonds (2.3257 (16) and 2.2979 (16) Å). While the Mn—O3/O4 bond lengths (2.3395 (14) and 2.3232 (14) Å) are almost equal, the Mn—O2 bond (2.4997 (15) Å) is considerably longer than the Mn—O1 bond (2.2425 (15) Å). The compound displays intermolecular O—H···O hydrogen-bond interactions (Table 1) to form various kinds of ring structures (8- and 28-membered ring with consideration of H-atoms) and cyclic water clusters (Fig. 3 and Fig. 4). The water clusters consist of a water tetramer (O3w/O1w^ii^/O6w^ii^/O5w^iii^) and two kinds of water hexamers (O2w/O1w/O6w/O2w^i^/O1w^i^/O6w^i^ and O1w/O2w/O3w/O1w^ii^/O2w^ii^/O3w^ii^), forming a polycyclic and one-dimensional chain structure along the *a* axis (Fig. 4). In the graph set notation for the structure, the 8-, 28-membered ring, and the water tetramer can be described by *R*_3_^3^(8), *R*_12_^12^(28), and *R*_4_^4^(8), respectively (Bernstein *et al.*, 1995). Both of the water hexamers can be represented by *R*_6_^6^(12), the basic binary graph set is C(8), and therefore the full designation is C(8)[*R*_6_^6^(12)].

### Experimental

A solution of Mn(CH_3_CO_2_)_2_.4H_2_O (0.42 g, 1.71 mmol) and *N*,*N*'-bis(2-pyridylmethylidene)cyclohexane-1,2-diamine (0.50 g, 1.71 mmol) in EtOH (20 ml) was stirred for 2 h at room temparature. After add of diethyl ether to the solution, the formed dark brown precipitate was removed by filtration. The solvent of the filtrate was evaporated, the residue washed with acetone and dried under vacuum, to give a yellow powder (0.38 g). Crystals suitable for X-ray analysis were obtained by slow evaporation from an MeOH solution. MS (FAB): m/*z* 406 (Mn(bpic)(CH_3_CO_2_)^+^); IR (KBr): 3471 cm^-1^ (broad).

### Refinement

H atoms were positioned geometrically and allowed to ride on their respective carrier atoms [C—H = 0.94 (aromatic CH), 0.97 (CH_3_), 0.98 (CH_2_) or 0.99 Å (CH) and *U*_iso_(H) = 1.2*U*_eq_(CH, CH_2_) or 1.5*U*_eq_(CH_3_)]. The H atoms of the solvent water molecules were located from difference maps then allowed to ride on their parent O atom in the final cycles of refinement.

### Crystal data

**Table:**

[Mn(C_2_H_3_O_2_)_2_(C_18_H_20_N_4_)]·6H_2_O	*Z* = 2
*M**_r_* = 573.50	*F*(000) = 606
Triclinic, *P*1	*D*_x_ = 1.355 Mg m^−^^3^
Hall symbol: -P 1	Mo *K*α radiation, λ = 0.71073 Å
*a* = 8.5124 (5) Å	Cell parameters from 5115 reflections
*b* = 11.6768 (7) Å	θ = 2.5–26.4°
*c* = 15.1971 (10) Å	µ = 0.53 mm^−^^1^
α = 79.049 (1)°	*T* = 243 K
β = 85.195 (1)°	Plate, yellow
γ = 71.484 (1)°	0.25 × 0.25 × 0.20 mm
*V* = 1405.83 (15) Å^3^	

### Data collection

**Table:**

Bruker SMART 1000 CCD diffractometer	5710 independent reflections
Radiation source: fine-focus sealed tube	4641 reflections with *I* > 2σ(*I*)
graphite	*R*_int_ = 0.015
φ and ω scans	θ_max_ = 26.4°, θ_min_ = 2.1°
Absorption correction: multi-scan (*SADABS*; Bruker, 2000)	*h* = −10→10
*T*_min_ = 0.824, *T*_max_ = 0.900	*k* = −14→11
11880 measured reflections	*l* = −19→15

### Refinement

**Table:**

Refinement on *F*^2^	Primary atom site location: structure-invariant direct methods
Least-squares matrix: full	Secondary atom site location: difference Fourier map
*R*[*F*^2^ > 2σ(*F*^2^)] = 0.040	Hydrogen site location: inferred from neighbouring sites
*wR*(*F*^2^) = 0.111	H-atom parameters constrained
*S* = 1.02	*w* = 1/[σ^2^(*F*_o_^2^) + (0.0678*P*)^2^] where *P* = (*F*_o_^2^ + 2*F*_c_^2^)/3
5710 reflections	(Δ/σ)_max_ < 0.001
348 parameters	Δρ_max_ = 0.44 e Å^−^^3^
0 restraints	Δρ_min_ = −0.20 e Å^−^^3^

### Special details

**Table:**

Geometry. All e.s.d.'s (except the e.s.d. in the dihedral angle between two l.s. planes) are estimated using the full covariance matrix. The cell e.s.d.'s are taken into account individually in the estimation of e.s.d.'s in distances, angles and torsion angles; correlations between e.s.d.'s in cell parameters are only used when they are defined by crystal symmetry. An approximate (isotropic) treatment of cell e.s.d.'s is used for estimating e.s.d.'s involving l.s. planes.
Refinement. Refinement of *F*^2^ against ALL reflections. The weighted *R*-factor *wR* and goodness of fit *S* are based on *F*^2^, conventional *R*-factors *R* are based on *F*, with *F* set to zero for negative *F*^2^. The threshold expression of *F*^2^ > σ(*F*^2^) is used only for calculating *R*-factors(gt) *etc*. and is not relevant to the choice of reflections for refinement. *R*-factors based on *F*^2^ are statistically about twice as large as those based on *F*, and *R*- factors based on ALL data will be even larger.

### Fractional atomic coordinates and isotropic or equivalent isotropic displacement parameters (Å^2^)

**Table:**

	*x*	*y*	*z*	*U*_iso_*/*U*_eq_	
Mn	0.10542 (3)	0.36835 (2)	0.246905 (18)	0.03241 (11)	
O1	−0.00485 (18)	0.21992 (14)	0.23912 (11)	0.0489 (4)	
O2	−0.17312 (19)	0.40053 (14)	0.18729 (11)	0.0521 (4)	
O3	0.30084 (18)	0.22868 (14)	0.34637 (10)	0.0485 (4)	
O4	0.33692 (17)	0.40494 (13)	0.29163 (10)	0.0447 (3)	
N1	0.2970 (2)	0.26036 (15)	0.14149 (11)	0.0376 (4)	
N2	0.1216 (2)	0.49712 (14)	0.11176 (11)	0.0376 (4)	
N3	−0.0140 (2)	0.56946 (14)	0.26365 (11)	0.0394 (4)	
N4	−0.0488 (2)	0.38102 (15)	0.38548 (11)	0.0395 (4)	
C1	0.3813 (3)	0.14166 (18)	0.15517 (15)	0.0430 (5)	
H1	0.3627	0.0930	0.2094	0.052*	
C2	0.4955 (3)	0.0858 (2)	0.09360 (16)	0.0472 (5)	
H2	0.5521	0.0013	0.1059	0.057*	
C3	0.5244 (3)	0.1557 (2)	0.01452 (16)	0.0488 (5)	
H3	0.6011	0.1200	−0.0284	0.059*	
C4	0.4383 (3)	0.2801 (2)	−0.00113 (14)	0.0442 (5)	
H4	0.4565	0.3304	−0.0545	0.053*	
C5	0.3251 (2)	0.32855 (18)	0.06336 (13)	0.0364 (4)	
C6	0.2242 (3)	0.45858 (18)	0.05085 (13)	0.0416 (5)	
H6	0.2361	0.5124	−0.0019	0.050*	
C7	0.0127 (3)	0.62390 (18)	0.10445 (14)	0.0421 (5)	
H7	−0.1019	0.6233	0.0986	0.051*	
C8	0.0499 (3)	0.71342 (19)	0.02540 (15)	0.0518 (6)	
H8A	0.0345	0.6888	−0.0306	0.062*	
H8B	0.1658	0.7111	0.0268	0.062*	
C9	−0.0627 (3)	0.8432 (2)	0.02776 (17)	0.0600 (6)	
H9A	−0.0326	0.8997	−0.0221	0.072*	
H9B	−0.1778	0.8473	0.0205	0.072*	
C10	−0.0483 (3)	0.8819 (2)	0.11493 (18)	0.0613 (7)	
H10A	−0.1237	0.9650	0.1156	0.074*	
H10B	0.0650	0.8831	0.1204	0.074*	
C11	−0.0909 (3)	0.79408 (19)	0.19376 (16)	0.0533 (6)	
H11A	−0.0782	0.8195	0.2499	0.064*	
H11B	−0.2067	0.7973	0.1904	0.064*	
C12	0.0206 (3)	0.66403 (18)	0.19347 (14)	0.0448 (5)	
H12	0.1359	0.6625	0.2003	0.054*	
C13	−0.1067 (3)	0.59486 (19)	0.33081 (14)	0.0476 (5)	
H13	−0.1608	0.6770	0.3365	0.057*	
C14	−0.1294 (3)	0.49444 (19)	0.40028 (14)	0.0428 (5)	
C15	−0.2256 (3)	0.5172 (2)	0.47669 (16)	0.0625 (7)	
H15	−0.2823	0.5981	0.4845	0.075*	
C16	−0.2375 (3)	0.4208 (2)	0.54085 (16)	0.0628 (7)	
H16	−0.3011	0.4344	0.5937	0.075*	
C17	−0.1552 (3)	0.3041 (2)	0.52677 (15)	0.0542 (6)	
H17	−0.1617	0.2360	0.5696	0.065*	
C18	−0.0623 (3)	0.2882 (2)	0.44841 (15)	0.0478 (5)	
H18	−0.0059	0.2078	0.4391	0.057*	
C19	−0.1417 (3)	0.2873 (2)	0.20639 (13)	0.0395 (5)	
C20	−0.2647 (3)	0.2269 (2)	0.18806 (15)	0.0492 (5)	
H20A	−0.3731	0.2876	0.1794	0.074*	
H20B	−0.2702	0.1633	0.2385	0.074*	
H20C	−0.2300	0.1908	0.1344	0.074*	
C21	0.3823 (2)	0.30202 (19)	0.34188 (13)	0.0399 (5)	
C22	0.5335 (3)	0.2686 (2)	0.39741 (16)	0.0576 (6)	
H22A	0.5028	0.2529	0.4604	0.086*	
H22B	0.5805	0.3357	0.3863	0.086*	
H22C	0.6146	0.1955	0.3813	0.086*	
O1W	0.2901 (2)	0.00775 (14)	0.43614 (12)	0.0567 (4)	
H1WA	0.3031	0.0826	0.4108	0.070 (8)*	
H1WB	0.2780	0.0170	0.4826	0.30 (4)*	
O2W	0.1964 (3)	0.0360 (3)	0.61170 (14)	0.0812 (6)	
H2WA	0.1671	0.1024	0.6022	0.13 (2)*	
H2WB	0.1013	0.0375	0.6368	0.139 (16)*	
O3W	0.4248 (2)	0.1469 (2)	0.63610 (18)	0.0914 (7)	
H3WA	0.5088	0.1076	0.6083	0.091 (11)*	
H3WB	0.4604	0.1624	0.6875	0.18 (2)*	
O4W	0.5080 (2)	0.56612 (16)	0.21655 (16)	0.0819 (6)	
H4WA	0.6103	0.5220	0.2098	0.085 (10)*	
H4WB	0.4635	0.5085	0.2389	0.103 (12)*	
O5W	0.4092 (3)	0.81922 (17)	0.22246 (14)	0.0772 (6)	
H5WA	0.4297	0.7432	0.2268	0.080 (10)*	
H5WB	0.3272	0.8234	0.2554	0.17 (2)*	
O6W	0.1096 (2)	−0.02700 (15)	0.30287 (12)	0.0592 (4)	
H6WA	0.0627	0.0545	0.2760	0.082 (9)*	
H6WB	0.1642	0.0000	0.3400	0.161 (18)*	

### Atomic displacement parameters (Å^2^)

**Table:**

	*U*^11^	*U*^22^	*U*^33^	*U*^12^	*U*^13^	*U*^23^
Mn	0.03681 (18)	0.02715 (17)	0.03107 (17)	−0.00713 (12)	0.00063 (12)	−0.00511 (11)
O1	0.0391 (8)	0.0549 (9)	0.0551 (9)	−0.0131 (7)	0.0003 (7)	−0.0183 (7)
O2	0.0579 (10)	0.0514 (10)	0.0519 (9)	−0.0249 (8)	0.0013 (7)	−0.0086 (7)
O3	0.0504 (9)	0.0487 (9)	0.0446 (9)	−0.0162 (7)	0.0017 (7)	−0.0035 (7)
O4	0.0431 (8)	0.0404 (8)	0.0479 (9)	−0.0090 (6)	−0.0052 (7)	−0.0058 (7)
N1	0.0417 (9)	0.0353 (9)	0.0366 (9)	−0.0122 (7)	0.0025 (7)	−0.0089 (7)
N2	0.0489 (10)	0.0310 (8)	0.0326 (9)	−0.0115 (7)	0.0006 (7)	−0.0065 (7)
N3	0.0495 (10)	0.0320 (9)	0.0340 (9)	−0.0082 (8)	−0.0002 (8)	−0.0068 (7)
N4	0.0417 (9)	0.0379 (9)	0.0348 (9)	−0.0052 (7)	−0.0004 (7)	−0.0092 (7)
C1	0.0497 (12)	0.0344 (11)	0.0450 (12)	−0.0122 (9)	0.0014 (10)	−0.0098 (9)
C2	0.0443 (12)	0.0395 (12)	0.0587 (14)	−0.0078 (10)	−0.0027 (10)	−0.0185 (10)
C3	0.0407 (12)	0.0544 (14)	0.0553 (14)	−0.0120 (10)	0.0079 (10)	−0.0274 (11)
C4	0.0466 (12)	0.0530 (13)	0.0375 (11)	−0.0198 (10)	0.0077 (9)	−0.0144 (9)
C5	0.0417 (11)	0.0382 (11)	0.0327 (10)	−0.0155 (9)	0.0019 (8)	−0.0104 (8)
C6	0.0556 (13)	0.0395 (11)	0.0316 (10)	−0.0190 (10)	0.0047 (9)	−0.0059 (8)
C7	0.0520 (13)	0.0342 (11)	0.0392 (11)	−0.0123 (9)	−0.0010 (9)	−0.0058 (8)
C8	0.0720 (16)	0.0378 (12)	0.0405 (12)	−0.0146 (11)	0.0032 (11)	−0.0006 (9)
C9	0.0719 (16)	0.0416 (13)	0.0589 (16)	−0.0143 (12)	0.0001 (12)	0.0035 (11)
C10	0.0750 (17)	0.0353 (12)	0.0701 (17)	−0.0155 (12)	0.0096 (13)	−0.0081 (11)
C11	0.0665 (15)	0.0360 (12)	0.0536 (14)	−0.0103 (11)	0.0073 (11)	−0.0120 (10)
C12	0.0553 (13)	0.0353 (11)	0.0421 (12)	−0.0117 (10)	0.0017 (10)	−0.0078 (9)
C13	0.0587 (14)	0.0331 (11)	0.0427 (12)	−0.0014 (10)	0.0058 (10)	−0.0116 (9)
C14	0.0481 (12)	0.0410 (11)	0.0343 (11)	−0.0042 (9)	0.0012 (9)	−0.0118 (9)
C15	0.0778 (18)	0.0511 (14)	0.0487 (14)	−0.0061 (13)	0.0186 (12)	−0.0176 (11)
C16	0.0758 (18)	0.0690 (17)	0.0383 (13)	−0.0157 (14)	0.0151 (12)	−0.0153 (12)
C17	0.0607 (15)	0.0579 (15)	0.0386 (12)	−0.0159 (12)	0.0033 (11)	−0.0013 (10)
C18	0.0547 (13)	0.0380 (11)	0.0436 (12)	−0.0076 (10)	0.0010 (10)	−0.0029 (9)
C19	0.0418 (12)	0.0518 (13)	0.0311 (10)	−0.0206 (10)	0.0081 (9)	−0.0150 (9)
C20	0.0476 (13)	0.0617 (14)	0.0465 (13)	−0.0258 (11)	0.0011 (10)	−0.0144 (11)
C21	0.0362 (11)	0.0473 (12)	0.0329 (10)	−0.0055 (9)	0.0035 (8)	−0.0138 (9)
C22	0.0445 (13)	0.0690 (16)	0.0508 (14)	−0.0004 (12)	−0.0108 (11)	−0.0150 (12)
O1W	0.0722 (11)	0.0445 (9)	0.0502 (10)	−0.0161 (8)	0.0028 (8)	−0.0054 (7)
O2W	0.0723 (14)	0.105 (2)	0.0708 (14)	−0.0352 (12)	0.0181 (11)	−0.0214 (12)
O3W	0.0600 (12)	0.0983 (16)	0.1221 (19)	−0.0099 (11)	0.0115 (12)	−0.0648 (15)
O4W	0.0647 (13)	0.0402 (10)	0.1278 (19)	−0.0123 (9)	0.0159 (11)	0.0025 (10)
O5W	0.0905 (15)	0.0564 (13)	0.0820 (14)	−0.0182 (10)	0.0101 (12)	−0.0188 (10)
O6W	0.0592 (10)	0.0523 (10)	0.0660 (11)	−0.0178 (8)	0.0009 (9)	−0.0097 (8)

### Geometric parameters (Å, °)

**Table:**

Mn—O1	2.2425 (15)	C9—H9B	0.9800
Mn—N3	2.2979 (16)	C10—C11	1.521 (3)
Mn—O4	2.3232 (14)	C10—H10A	0.9800
Mn—N2	2.3257 (16)	C10—H10B	0.9800
Mn—O3	2.3395 (14)	C11—C12	1.514 (3)
Mn—N4	2.3899 (17)	C11—H11A	0.9800
Mn—N1	2.4166 (16)	C11—H11B	0.9800
Mn—O2	2.4997 (15)	C12—H12	0.9900
O1—C19	1.261 (2)	C13—C14	1.472 (3)
O2—C19	1.244 (3)	C13—H13	0.9400
O3—C21	1.251 (3)	C14—C15	1.382 (3)
O4—C21	1.258 (2)	C15—C16	1.366 (3)
N1—C1	1.328 (3)	C15—H15	0.9400
N1—C5	1.347 (2)	C16—C17	1.369 (3)
N2—C6	1.263 (3)	C16—H16	0.9400
N2—C7	1.465 (2)	C17—C18	1.382 (3)
N3—C13	1.258 (3)	C17—H17	0.9400
N3—C12	1.466 (3)	C18—H18	0.9400
N4—C18	1.330 (3)	C19—C20	1.504 (3)
N4—C14	1.336 (3)	C20—H20A	0.9700
C1—C2	1.387 (3)	C20—H20B	0.9700
C1—H1	0.9400	C20—H20C	0.9700
C2—C3	1.370 (3)	C21—C22	1.503 (3)
C2—H2	0.9400	C22—H22A	0.9700
C3—C4	1.388 (3)	C22—H22B	0.9700
C3—H3	0.9400	C22—H22C	0.9700
C4—C5	1.384 (3)	O1W—H1WA	0.922
C4—H4	0.9400	O1W—H1WB	0.728
C5—C6	1.473 (3)	O2W—H2WA	0.724
C6—H6	0.9400	O2W—H2WB	0.862
C7—C8	1.518 (3)	O3W—H3WA	0.846
C7—C12	1.526 (3)	O3W—H3WB	0.929
C7—H7	0.9900	O4W—H4WA	0.868
C8—C9	1.520 (3)	O4W—H4WB	0.875
C8—H8A	0.9800	O5W—H5WA	0.839
C8—H8B	0.9800	O5W—H5WB	0.818
C9—C10	1.505 (4)	O6W—H6WA	0.933
C9—H9A	0.9800	O6W—H6WB	0.916
			
O1—Mn—N3	131.76 (6)	C9—C8—H8A	109.4
O1—Mn—O4	143.52 (5)	C7—C8—H8B	109.4
N3—Mn—O4	81.25 (6)	C9—C8—H8B	109.4
O1—Mn—N2	115.12 (6)	H8A—C8—H8B	108.0
N3—Mn—N2	70.09 (6)	C10—C9—C8	110.9 (2)
O4—Mn—N2	88.15 (6)	C10—C9—H9A	109.5
O1—Mn—O3	89.34 (5)	C8—C9—H9A	109.5
N3—Mn—O3	122.24 (6)	C10—C9—H9B	109.5
O4—Mn—O3	55.61 (5)	C8—C9—H9B	109.5
N2—Mn—O3	134.37 (6)	H9A—C9—H9B	108.1
O1—Mn—N4	84.05 (6)	C9—C10—C11	110.6 (2)
N3—Mn—N4	69.42 (6)	C9—C10—H10A	109.5
O4—Mn—N4	97.39 (6)	C11—C10—H10A	109.5
N2—Mn—N4	137.68 (6)	C9—C10—H10B	109.5
O3—Mn—N4	79.35 (5)	C11—C10—H10B	109.5
O1—Mn—N1	79.49 (5)	H10A—C10—H10B	108.1
N3—Mn—N1	136.20 (6)	C12—C11—C10	110.97 (19)
O4—Mn—N1	84.34 (5)	C12—C11—H11A	109.4
N2—Mn—N1	68.30 (6)	C10—C11—H11A	109.4
O3—Mn—N1	80.28 (5)	C12—C11—H11B	109.4
N4—Mn—N1	153.85 (6)	C10—C11—H11B	109.4
O1—Mn—O2	54.45 (5)	H11A—C11—H11B	108.0
N3—Mn—O2	81.85 (6)	N3—C12—C11	116.19 (18)
O4—Mn—O2	162.02 (5)	N3—C12—C7	106.13 (16)
N2—Mn—O2	80.34 (5)	C11—C12—C7	111.00 (17)
O3—Mn—O2	140.89 (5)	N3—C12—H12	107.7
N4—Mn—O2	82.32 (5)	C11—C12—H12	107.7
N1—Mn—O2	103.87 (5)	C7—C12—H12	107.7
C19—O1—Mn	97.99 (12)	N3—C13—C14	119.20 (18)
C19—O2—Mn	86.39 (12)	N3—C13—H13	120.4
C21—O3—Mn	91.80 (12)	C14—C13—H13	120.4
C21—O4—Mn	92.38 (12)	N4—C14—C15	122.6 (2)
C1—N1—C5	117.49 (17)	N4—C14—C13	115.85 (18)
C1—N1—Mn	125.96 (14)	C15—C14—C13	121.57 (19)
C5—N1—Mn	116.50 (12)	C16—C15—C14	119.3 (2)
C6—N2—C7	123.33 (17)	C16—C15—H15	120.3
C6—N2—Mn	120.53 (13)	C14—C15—H15	120.3
C7—N2—Mn	116.07 (12)	C15—C16—C17	118.8 (2)
C13—N3—C12	122.54 (17)	C15—C16—H16	120.6
C13—N3—Mn	119.87 (14)	C17—C16—H16	120.6
C12—N3—Mn	117.58 (12)	C16—C17—C18	118.8 (2)
C18—N4—C14	117.34 (18)	C16—C17—H17	120.6
C18—N4—Mn	127.12 (14)	C18—C17—H17	120.6
C14—N4—Mn	115.54 (13)	N4—C18—C17	123.2 (2)
N1—C1—C2	123.4 (2)	N4—C18—H18	118.4
N1—C1—H1	118.3	C17—C18—H18	118.4
C2—C1—H1	118.3	O2—C19—O1	121.13 (19)
C3—C2—C1	118.9 (2)	O2—C19—C20	120.55 (19)
C3—C2—H2	120.5	O1—C19—C20	118.29 (19)
C1—C2—H2	120.5	C19—C20—H20A	109.5
C2—C3—C4	118.8 (2)	C19—C20—H20B	109.5
C2—C3—H3	120.6	H20A—C20—H20B	109.5
C4—C3—H3	120.6	C19—C20—H20C	109.5
C5—C4—C3	118.7 (2)	H20A—C20—H20C	109.5
C5—C4—H4	120.6	H20B—C20—H20C	109.5
C3—C4—H4	120.6	O3—C21—O4	120.18 (18)
N1—C5—C4	122.72 (18)	O3—C21—C22	120.3 (2)
N1—C5—C6	114.99 (17)	O4—C21—C22	119.5 (2)
C4—C5—C6	122.28 (18)	C21—C22—H22A	109.5
N2—C6—C5	119.34 (18)	C21—C22—H22B	109.5
N2—C6—H6	120.3	H22A—C22—H22B	109.5
C5—C6—H6	120.3	C21—C22—H22C	109.5
N2—C7—C8	115.54 (18)	H22A—C22—H22C	109.5
N2—C7—C12	106.62 (15)	H22B—C22—H22C	109.5
C8—C7—C12	111.62 (17)	H1WA—O1W—H1WB	98
N2—C7—H7	107.6	H2WA—O2W—H2WB	88
C8—C7—H7	107.6	H3WA—O3W—H3WB	108
C12—C7—H7	107.6	H4WA—O4W—H4WB	100
C7—C8—C9	111.09 (19)	H5WA—O5W—H5WB	91
C7—C8—H8A	109.4	H6WA—O6W—H6WB	88
			
N3—Mn—O1—C19	28.31 (15)	N3—Mn—N4—C18	−177.26 (18)
O4—Mn—O1—C19	178.17 (11)	O4—Mn—N4—C18	−99.54 (17)
N2—Mn—O1—C19	−56.78 (13)	N2—Mn—N4—C18	165.06 (16)
O3—Mn—O1—C19	163.13 (12)	O3—Mn—N4—C18	−46.69 (17)
N4—Mn—O1—C19	83.77 (12)	N1—Mn—N4—C18	−7.3 (2)
N1—Mn—O1—C19	−116.63 (13)	O2—Mn—N4—C18	98.60 (17)
O2—Mn—O1—C19	−1.03 (11)	O1—Mn—N4—C14	−136.26 (15)
O1—Mn—O2—C19	1.03 (11)	N3—Mn—N4—C14	2.72 (14)
N3—Mn—O2—C19	−157.30 (13)	O4—Mn—N4—C14	80.45 (15)
O4—Mn—O2—C19	−177.43 (15)	N2—Mn—N4—C14	−14.95 (19)
N2—Mn—O2—C19	131.64 (13)	O3—Mn—N4—C14	133.30 (15)
O3—Mn—O2—C19	−24.61 (16)	N1—Mn—N4—C14	172.66 (14)
N4—Mn—O2—C19	−87.12 (12)	O2—Mn—N4—C14	−81.41 (15)
N1—Mn—O2—C19	67.00 (12)	C5—N1—C1—C2	0.1 (3)
O1—Mn—O3—C21	170.16 (12)	Mn—N1—C1—C2	−176.94 (15)
N3—Mn—O3—C21	−48.56 (14)	N1—C1—C2—C3	0.3 (3)
O4—Mn—O3—C21	0.94 (11)	C1—C2—C3—C4	0.0 (3)
N2—Mn—O3—C21	44.52 (15)	C2—C3—C4—C5	−0.7 (3)
N4—Mn—O3—C21	−105.77 (12)	C1—N1—C5—C4	−0.9 (3)
N1—Mn—O3—C21	90.71 (12)	Mn—N1—C5—C4	176.47 (15)
O2—Mn—O3—C21	−169.22 (11)	C1—N1—C5—C6	178.17 (18)
O1—Mn—O4—C21	−19.26 (17)	Mn—N1—C5—C6	−4.5 (2)
N3—Mn—O4—C21	138.47 (13)	C3—C4—C5—N1	1.2 (3)
N2—Mn—O4—C21	−151.39 (12)	C3—C4—C5—C6	−177.80 (19)
O3—Mn—O4—C21	−0.93 (11)	C7—N2—C6—C5	−178.27 (18)
N4—Mn—O4—C21	70.72 (12)	Mn—N2—C6—C5	4.6 (3)
N1—Mn—O4—C21	−83.02 (12)	N1—C5—C6—N2	0.1 (3)
O2—Mn—O4—C21	158.63 (16)	C4—C5—C6—N2	179.21 (19)
O1—Mn—N1—C1	−55.50 (16)	C6—N2—C7—C8	−10.0 (3)
N3—Mn—N1—C1	162.74 (15)	Mn—N2—C7—C8	167.23 (15)
O4—Mn—N1—C1	91.65 (16)	C6—N2—C7—C12	−134.7 (2)
N2—Mn—N1—C1	−178.08 (18)	Mn—N2—C7—C12	42.56 (19)
O3—Mn—N1—C1	35.63 (16)	N2—C7—C8—C9	−176.28 (19)
N4—Mn—N1—C1	−3.6 (2)	C12—C7—C8—C9	−54.2 (3)
O2—Mn—N1—C1	−104.60 (16)	C7—C8—C9—C10	56.3 (3)
O1—Mn—N1—C5	127.40 (14)	C8—C9—C10—C11	−57.9 (3)
N3—Mn—N1—C5	−14.36 (17)	C9—C10—C11—C12	57.8 (3)
O4—Mn—N1—C5	−85.44 (13)	C13—N3—C12—C11	−13.9 (3)
N2—Mn—N1—C5	4.82 (13)	Mn—N3—C12—C11	165.37 (15)
O3—Mn—N1—C5	−141.47 (14)	C13—N3—C12—C7	−137.8 (2)
N4—Mn—N1—C5	179.31 (13)	Mn—N3—C12—C7	41.4 (2)
O2—Mn—N1—C5	78.31 (14)	C10—C11—C12—N3	−176.85 (19)
O1—Mn—N2—C6	−71.18 (17)	C10—C11—C12—C7	−55.5 (3)
N3—Mn—N2—C6	161.05 (17)	N2—C7—C12—N3	−51.9 (2)
O4—Mn—N2—C6	79.68 (16)	C8—C7—C12—N3	−178.90 (18)
O3—Mn—N2—C6	44.99 (19)	N2—C7—C12—C11	−178.96 (17)
N4—Mn—N2—C6	178.64 (14)	C8—C7—C12—C11	54.0 (2)
N1—Mn—N2—C6	−4.96 (15)	C12—N3—C13—C14	−177.34 (18)
O2—Mn—N2—C6	−114.19 (16)	Mn—N3—C13—C14	3.4 (3)
O1—Mn—N2—C7	111.51 (14)	C18—N4—C14—C15	−0.8 (3)
N3—Mn—N2—C7	−16.27 (13)	Mn—N4—C14—C15	179.20 (18)
O4—Mn—N2—C7	−97.64 (14)	C18—N4—C14—C13	177.84 (19)
O3—Mn—N2—C7	−132.33 (13)	Mn—N4—C14—C13	−2.1 (2)
N4—Mn—N2—C7	1.33 (18)	N3—C13—C14—N4	−0.7 (3)
N1—Mn—N2—C7	177.72 (15)	N3—C13—C14—C15	177.9 (2)
O2—Mn—N2—C7	68.49 (14)	N4—C14—C15—C16	1.1 (4)
O1—Mn—N3—C13	57.80 (19)	C13—C14—C15—C16	−177.4 (2)
O4—Mn—N3—C13	−104.62 (17)	C14—C15—C16—C17	−0.9 (4)
N2—Mn—N3—C13	164.18 (18)	C15—C16—C17—C18	0.4 (4)
O3—Mn—N3—C13	−65.21 (19)	C14—N4—C18—C17	0.3 (3)
N4—Mn—N3—C13	−3.27 (16)	Mn—N4—C18—C17	−179.74 (17)
N1—Mn—N3—C13	−176.88 (15)	C16—C17—C18—N4	−0.1 (4)
O2—Mn—N3—C13	81.55 (17)	Mn—O2—C19—O1	−1.75 (19)
O1—Mn—N3—C12	−121.46 (14)	Mn—O2—C19—C20	−179.73 (18)
O4—Mn—N3—C12	76.12 (14)	Mn—O1—C19—O2	2.0 (2)
N2—Mn—N3—C12	−15.08 (14)	Mn—O1—C19—C20	179.99 (15)
O3—Mn—N3—C12	115.53 (14)	Mn—O3—C21—O4	−1.7 (2)
N4—Mn—N3—C12	177.47 (15)	Mn—O3—C21—C22	177.48 (17)
N1—Mn—N3—C12	3.86 (18)	Mn—O4—C21—O3	1.7 (2)
O2—Mn—N3—C12	−97.71 (14)	Mn—O4—C21—C22	−177.47 (17)
O1—Mn—N4—C18	43.76 (17)		

### Hydrogen-bond geometry (Å, °)

**Table:**

*D*—H···*A*	*D*—H	H···*A*	*D*···*A*	*D*—H···*A*
O1W—H1WA···O3	0.922	1.793	2.705 (2)	169
O1W—H1WB···O2W	0.728	2.054	2.770 (3)	168
O2W—H2WA···O3W	0.724	2.523	2.738 (3)	100
O2W—H2WB···O6W^i^	0.862	1.975	2.835 (3)	176
O3W—H3WA···O1W^ii^	0.846	1.972	2.807 (3)	169
O3W—H3WB···O5W^iii^	0.929	1.915	2.821 (3)	165
O4W—H4WA···O2^iv^	0.868	1.986	2.845 (2)	170
O4W—H4WB···O4	0.875	1.892	2.759 (2)	171
O5W—H5WA···O4W	0.839	1.993	2.821 (3)	169
O5W—H5WB···O6W^v^	0.818	2.281	2.937 (3)	138
O6W—H6WA···O1	0.933	1.820	2.741 (2)	169
O6W—H6WB···O1W	0.916	1.915	2.803 (3)	163

Symmetry codes: (i) −*x*, −*y*, −*z*+1; (ii) −*x*+1, −*y*, −*z*+1; (iii) −*x*+1, −*y*+1, −*z*+1; (iv) *x*+1, *y*, *z*; (v) *x*, *y*+1, *z*.
